# Prestorage High CO_2_ and 1-MCP Treatment Reduce Chilling Injury, Prolong Storability, and Maintain Sensory Qualities and Antioxidant Activities of “Madoka” Peach Fruit

**DOI:** 10.3389/fnut.2022.903352

**Published:** 2022-05-18

**Authors:** Shimeles Tilahun, Min Jae Jeong, Han Ryul Choi, Min Woo Baek, Jin Sung Hong, Cheon Soon Jeong

**Affiliations:** ^1^Agriculture and Life Science Research Institute, Kangwon National University, Chuncheon, South Korea; ^2^Department of Horticulture and Plant Sciences, Jimma University, Jimma, Ethiopia; ^3^Department of Horticulture, Kangwon National University, Chuncheon, South Korea; ^4^Interdisciplinary Program in Smart Agriculture, Kangwon National University, Chuncheon, South Korea; ^5^Department of Applied Biology, Kangwon National University, Chuncheon, South Korea

**Keywords:** peach [*Prunus perica* (L) Batsch], cold storage (CS), chilling injury (CI), CI index, sensory qualities, antioxidant activities

## Abstract

Cold storage is widely used to prolong the storability of peach fruit. However, prolonged storage at low temperatures results in chilling injury (CI) in some susceptible peach cultivars during or after cold storage. Prestorage high CO_2_ and 1-methylcyclopropene (1-MCP) treatments are among the methods reported to alleviate CI and maintain the firmness of peach fruit. Hence, this study investigated CI, ripening-related physicochemical parameters, sensory qualities, total phenolics and flavonoids, and antioxidant activities of “Madoka” peach fruit to observe the effectiveness of prestorage treatment with high CO_2_ and 1-MCP during the storage at 0 and 5°C. Based on the CI index, control fruits were acceptable for marketing up to 20 and 16 days of storage at 0 and 5°C, respectively, while the treated fruits could be marketable up to 28 days of storage. The results of firmness and firmness-related parameters [pectin content and polygalacturonase (PG) activity] also revealed that both high CO_2_ and 1-MCP treatments were effective in delaying the ripening process of Madoka peach, and the storage at 0°C showed better results than at 5°C. However, based on the overall sensory evaluation results, the treated and control fruits were acceptable for marketing up to 20 and 12 days of storage, respectively, in both storage conditions. After deciding on fruit marketability based on the combined objective postharvest quality parameters and subjective sensory qualities, we analyzed the changes in total phenolics, flavonoids, and antioxidant activities at harvest, on the 12 and 20th days of cold storage. Storage of Madoka peach at 0°C maintained total phenolics, flavonoids, and antioxidant activities regardless of prestorage treatment with high CO_2_ and 1-MCP. In summary, storing Madoka peach fruit at 0°C after treating it with 30% CO_2_ for 6 h or 0.5 μl L^–1^ 1-MCP for 24 h reduces CI, prolongs storability, and maintains sensory quality and antioxidant properties.

## Introduction

Peach [*Prunus persica* (L.) Batsch] is a healthy and delicious fruit that belongs to the Rosaceae family and is cultivated in temperate regions, between 30 and 45° of latitude, in both hemispheres (1). Peach is the third most important temperate tree fruit in the world, after apple and pear, and more than 90% of its production is for the fresh market (1). Nowadays, China leads the world’s peach production followed by Italy, Spain, the United States, and Greece (1). According to food and agriculture organization corporate statistical database (FAOSTAT) (2), 24.57 million Mt of peaches and nectarines were produced per year in 2020, covering 1.49 million ha, out of which the Republic of Korea took a share of 189,058 Mt (2). Peaches are a good source of bioactive compounds, such as phenolics, carotenoids, anthocyanin, and vitamins, making them ideal for a healthy diet against various chronic diseases due to their ability to scavenge reactive oxygen species produced in human blood plasma (3–5).

About 30 different cultivars of peaches and nectarines have been cultivated in Korea (6). Nectarines are denser as their cells have smaller intercellular spaces than peaches (7), and the peel (exocarp) of the fruit is pubescent in standard peaches or glabrous as nectarines (1). Peaches and nectarines are mainly categorized into melting and non-melting types based on flesh texture, according to firmness and pattern of softening, and they are strongly affected by cell wall composition and calcium content (1, 7). The melting type, including Madoka (the cultivar we used for this study), is the most common type that softens quickly along with physiological ripening (3).

Fresh peaches ripen and deteriorate quickly at ambient temperature. Therefore, cold storage alone or in combination with modified atmospheres and controlled atmospheres is the most effective way to slow down the product deterioration in terms of the consumer perception and nutritional value by reducing their overall metabolism which in turn delays ripening and senescence (8). However, prolonged storage at low-temperature results in chilling injury (CI) symptoms in some susceptible peach cultivars during or after cold storage (3, 5), especially with storage temperatures ranging from 2 to 8°C than those stored at 0°C (7). Mealy flesh (soft and dry, with no juice) or leathered flesh (hard textured, with no juice), with or without browning and bleeding in the flesh, is a symptom of CI (7) that affects its marketability. Hence, the methods to ameliorate CI can essentially contribute to the fruit industry.

Peach fruit’s maximum storage life of 3 weeks or more can be achieved near or below 0°C but above their freezing point depending on the soluble solids content of the peach fruit cultivars (7). In addition, Choi et al. (3) and Jin et al. (9) reported that treating peach fruit with 0.5 μL L^–1^ 1-methylcyclopropene (1-MCP) can prevent CI. Similarly, a significant reduction in CI and softening of peach fruit was reported following prestorage 30% CO_2_ treatment for 6 h (3, 6). Although the above studies have reported the effect of storage temperature and prestorage1-MCP and high CO_2_ treatment on the CI and physicochemical characteristics of peach fruit, the data on sensory qualities and changes in antioxidant activities are insufficient.

Fruit physiology and biochemistry are altered by cold storage, resulting in compositional changes in carbon- and nitrogen-related metabolisms and compounds (8). On the other hand, secondary metabolites are the compounds that contribute to fruit defense, aroma, and pigmentation (10). Therefore, the use of cold storage should take into account its effects on the most important secondary metabolites, such as the total phenolics and flavonoids. Furthermore, antioxidant activity indicates cumulative or synergistic effects of secondary metabolites (10). Hence, this study investigated CI, ripening-related physicochemical parameters, sensory qualities, the total phenolics and flavonoids, and antioxidant activities of Madoka peach fruit to observe the effectiveness of prestorage treatment with high CO_2_ and 1-MCP during the storage at 0 and 5°C.

## Materials and Methods

### Plant Material, Treatments, and Cold Storage

For this experiment, the fruit of Madoka peach cultivar, one of the commonly grown cultivars around Chuncheon, Korea was selected and harvested at commercial maturity (when the background color changes from green to cream color, firmness ranging from 61 to 65 N, and total soluble solids (TSS) ranging from 7.5 to 9%) on 20 September, 2020. The fruits were transported immediately to the postharvest laboratory at the Department of Horticultural Sciences, Kangwon National University. Uniform fruits free of defects were carefully selected and kept at 8°C for 3 h to remove field heat. The treatment groups were treated separately with 0.5 μL L^–1^ 1-MCP (Smart Fresh, Agro fresh Korea Ltd., Seoul, South Korea) for 24 h and 30% CO_2_ for 6 h in a sealed 242.64 L acrylic chamber at 0 and 5°C with 90–95% RH (3). The control fruits were treated under similar conditions without CO_2_ and 1-MCP treatment. Air in the sealed containers was ventilated and distributed by a fan (Coolertec CT8025L12RA-3P, Zhengzhou, China). A total of 6 containers were used for the treatments and control, 80 fruits were placed in each container. The treated fruits were stored at 0 and 5°C for 28 days. Hence, the samples were categorized as fruits at commercial harvest (control), and samples from the stored fruits at 4 days intervals [cold stored (CS), 1-MCP + CS, and CO_2_ + CS] for both 0 and 5°C. The data for firmness, weight loss, respiration rate, ethylene production rate, TSS, titratable acidity (TA), and sensory qualities were recorded at harvest and at 4 days intervals during the storage period. The samples of fruit flesh were also taken in three replicates using three fruits for each replicate on each experiment day. The samples were separated into two, frozen by liquid nitrogen, and stored in a deep freezer (–80°C) until analysis (3). One part of the frozen triplicate samples was used for the analysis of pectin content and PG activity. Then, the second part of the samples were freeze-dried with a vacuum freeze dryer (FDT-8650, Operon, South Korea), and the dried samples were ground to powder. The powdered samples were filtered with 40-μm mesh, packed in LDPE pouches, and then stored at –20°C until extraction (11). Subsequently, a quantitative analysis of total phenolics, total flavonoids, and antioxidant activities was carried out using freeze-dried samples of the pulp.

### Chilling Injury Index, Firmness, Pectin Content, and Polygalacturonase Activity

The CI was assessed visually, and the CI index was calculated according to the method described by Lee (6) based on the score of CI symptoms on a scale from 0 (no injury) to 5 (very severe).


$$\mathrm{CI}\,\mathrm{index}=\sum_{k=5}^{n}\frac{\left(C\,I\,l\,e\,v\,e\,l\right)*\left(\mathrm{Number}\,\mathrm{of}\,\mathrm{fruits}\,\mathrm{at}\,\mathrm{this}\,\mathrm{level}\right)}{T\,o\,t\,a\,l\,n\,u\,m\,b\,e\,r\,o\,f\,f\,r\,u\,i\,t\,s}$$

The peach fruit firmness was measured by a Rheometer (Sun Scientific Co. Ltd., Tokyo, Japan) from nine fruits, two measurements per fruit, by a puncture at the equator with a maximum force of 10 kg and an 8-mm diameter round stainless steel probe with a flat end, as stated by Tilahun et al. (12). The PG activity and quantification of pectin were made in three replicates following the methods described by Seo et al. (13).

### Respiration and Ethylene Production Rates

Respiration and ethylene production rates of peach fruit were measured and expressed as described by Belew et al. (14).

### Changes in Total Soluble Solids, Titratable Acidity, Brix to Acid Ratio, and Sensory Evaluation

The SSC and TA were measured from nine sample peach fruits according to Tilahun et al. (15). A digital refractometer (Atago Co., Ltd., Tokyo, Japan) was used to measure the TSS at 20°C. Fruit juice was diluted (1 ml juice: 19 ml distilled water) and titration of the diluted juice was done by DL22 Food and Beverage Analyzer (Mettler Toledo Ltd., Zurich, Switzerland) with 0.1 N⋅NaOH up to pH 8.1 to obtain TA and the result was expressed in mg 100 g^–1^. The BAR (Brix to Acid Ratio) was determined by dividing the TSS with TA (16).

The overall acceptability of kiwi fruit during the ripening period was evaluated as the mean value of the subjective scale made by 10 trained graduate students for flavor, color, sweetness, texture, sponginess, and overall sensory quality according to a subjective scale by Seo et al. (13) from bad (1) to excellent (5).

### Total Phenolics and Flavonoids

The total phenolics and flavonoids contents of freeze-dried peach fruit samples were measured in triplicate using the methodology implemented previously in our laboratory and described by Baek et al. (11). For the total phenolics analysis, ethanolic extract (1 mg ml^–1^) or standard was mixed with 1 ml of 10% Folin–Ciocalteu’s phenol reagent and 1 ml of 2% sodium carbonate solution. The absorbance was measured at 750 nm using a microplate reader (Spectramax i3, Molecular Devices, Sunnyvale, CA, United States) after incubation of the samples at ambient temperature for 90 min in dark. A comparison of the measurement to the calibration curve of gallic acid was made, and the results were expressed as milligrams of gallic acid equivalents (GAE) per gram of sample (mg GAE g^–1^). For total flavonoids analysis, ethanolic extract (1 mg ml^–1^) of the extract was mixed with 1.5 ml of ethanol, 0.1 ml of 10% aluminum nitrite solution, 0.1 ml of 1-M potassium acetate solution, and 2.8 ml distilled water. The mixture was stirred and allowed to react for 30 min. Then, the absorbance was measured at 415 nm using a microplate reader Spectramax i3, Molecular Devices, Sunnyvale, CA, United States). The measurements were compared to a quercetin (QE) calibration curve, and the results were expressed as milligrams of QE per gram of sample (mg QE g^–1^).

### Antioxidant Activities

Freeze-dried and ground peach fruit samples were extracted using the methodology described by Baek et al. (11), which had previously been implemented in our laboratory. The 2,2-di-phenyl-1-picrylhydrazyl (DPPH) radical scavenging capacity, Trolox equivalent antioxidant capacity (ABTS), and ferric reducing antioxidant power (FRAP) were measured in triplicate according to Baek et al. (11). The reducing power assay was also performed in triplicate according to the method reported by Choi et al. (17).

### Statistical Analysis

The experiment was conducted in a completely randomized design. The data were subjected to analysis of variance (ANOVA) to determine the significance of differences between treatments at *p* < 0.05 using SAS statistical software (SAS/STAT ^®^ 9.1; SAS Institute Inc., Cary, NC, United States). Duncan’s multiple range test was performed to observe differences between the treatment means.

## Results

### Chilling Injury, Firmness, Total Pectin, and Polygalacturonase Activity

**FIGURE 1:**
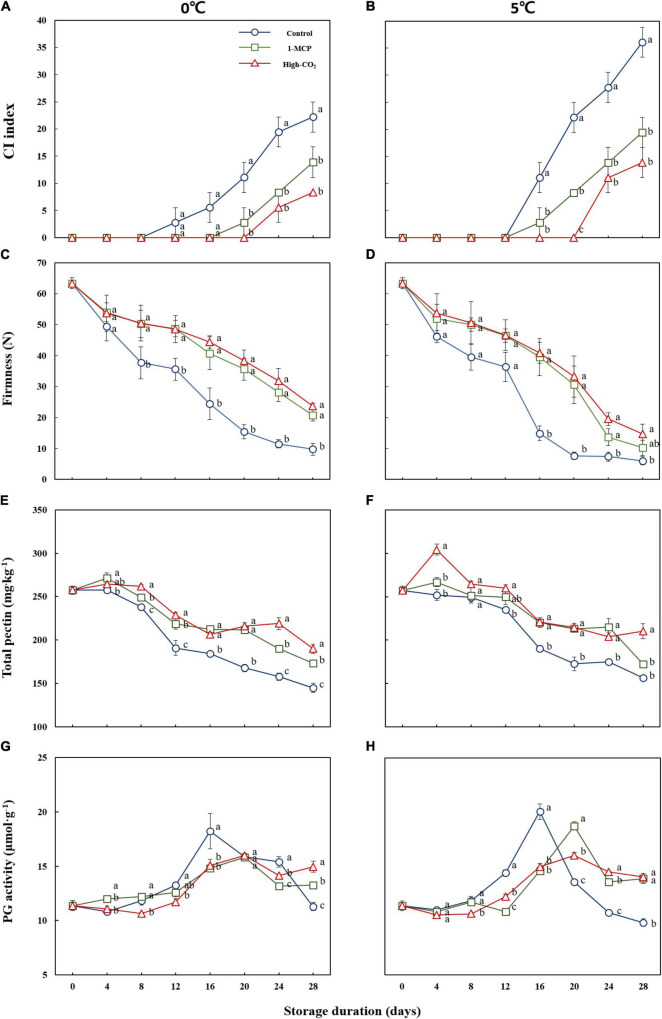
CI index **(A,B)**, firmness **(C,D)**, total pectin **(E,F)**, and PG activity **(G,H)** of Madoka peach fruit at harvest and during storage up to 28 days at 0 and 5°C with or without prestorage treatment of CO2 and 1-MCP.

The results of this study showed CI in the control fruit on the 12th and 16th day of the storage at 0 and 5°C, respectively. Also, the CI index progressed fast in the control fruit and attained 36.07% at 5°C as compared to 22.21% at 0°C on the 28th days. On the other hand, prestorage treatment of Madoka peach fruit with high CO_2_ and 1-MCP delayed the onset of CI and reduced its severity throughout the storage period at both 0 and 5°C (Figures 1A,B). As shown in Figure 1, prestorage high CO_2_ treatment delayed the onset of CI, and CI symptoms were not observed up to the 20th day of the storage at both 0 and 5°C. Similarly, prestorage 1-MCP treatment delayed the onset of CI up to 16 days at 0°C and reduced the severity throughout the storage period at both 0 and 5°C compared to the control.

Prestorage high CO_2_ and 1-MCP treatments were also effective in delaying the ripening process of Madoka peach fruit during the storage at both 0 and 5°C as observed on the firmness (Figures 1C,D). The firmness of the fruit at harvest was 63.32 N, and decreased to 9.74 and 5.98 N on the 28th day for the control fruit stored at 0 and 5°C, respectively. In contrast, high CO_2_-treated fruits showed a slow reducing trend and attained 23.73 and 14.62 N on the 28th day at 0 and 5°C, respectively. The 1-MCP-treated fruits also reduced to 20.72 and 10.22 N on the 28th day at 0 and 5°C, respectively. Generally, the results revealed that both high CO_2_ and 1-MCP treatments have statistically the same effects in maintaining the firmness of Madoka peach fruits during the storage at both 0 and 5°C (Figures 1C,D). However, significant differences in firmness were observed between the control and treatment groups starting from the 8th day at 0°C while the difference was observed after the 16th day at 5°C.

The total pectin content followed the same pattern as firmness and higher pectin contents were recorded from the treated fruits than the control throughout the storage period at both 0 and 5°C (Figures 1E,F). However, the PG activity was higher in control fruits than the treated fruits and the peak was attained earlier than the treated fruits on the 16th d at both 0 and 5°C. After reaching the peak, gradual reducing trends of PG activity were observed at 0°C while fast reducing trends were seen at 5°C (Figures 1G,H).

### Respiration and Ethylene Production Rates

**FIGURE 2:**
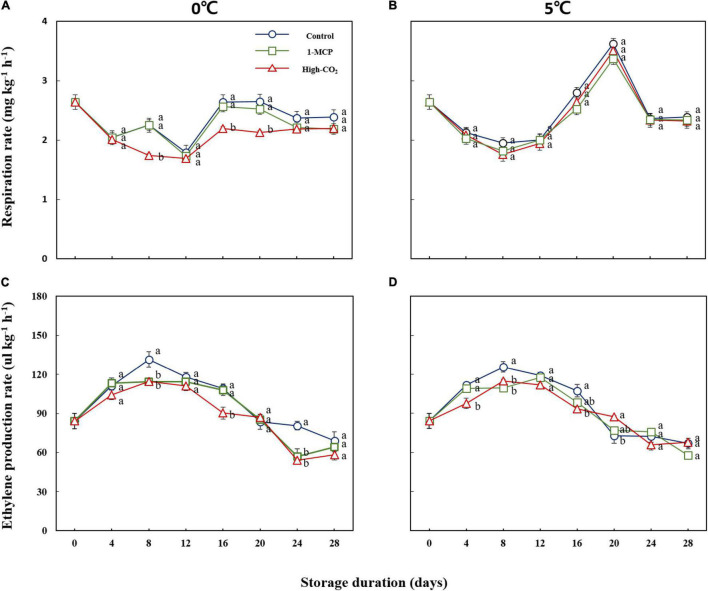
Respiration **(A,B)** and ethylene production rates **(C,D)** of Madoka peach fruit at harvest and during storage up to 28 days at 0 and 5° with or without prestorage treatment of CO2 and 1-MCP.

The effect of prestorage high CO_2_ and 1-MCP treatment on respiration and ethylene production rates was presented in Figures 2A,B. Significantly lower respiration rate was observed in prestorage high CO_2_ and 1-MCP-treated fruits at 0°C but there was no significant difference in respiration rate between the control and treated groups at 5°C. However, higher peaks of respiration rate for the control and treatment groups were observed at 5°C as compared to 0°C, implying the higher influence of storage temperature over prestorage treatments on the respiration rate of peach fruit. Besides, significantly higher ethylene production was observed in the control as compared to the treated groups and the peaks were attained on the 8th day of storage at both 0 and 5°C (Figures 2C,D).

### The Total Soluble Solids, Titratable Acidity, and Brix to Acid Ratio and Overall Sensory Evaluation

**FIGURE 3:**
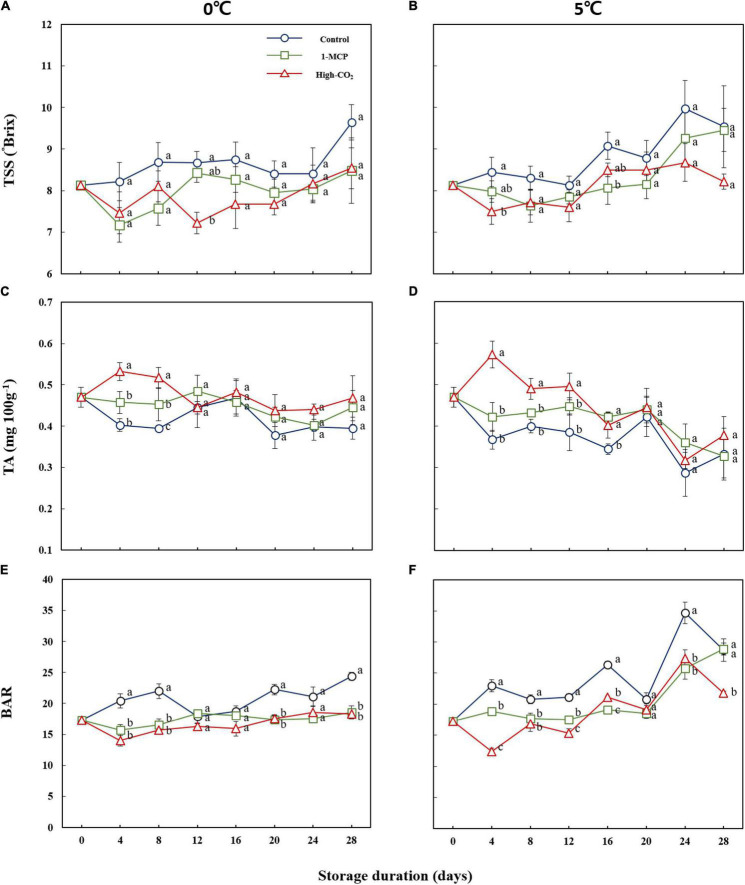
The TSS **(A,B)**, TA **(C,D)**, and BAR **(E,F)** of Madoka peach fruit at harvest and during storage up to 28 days at 0 and 5°C with or without prestorage treatment of CO2 and 1-MCP.

In this study, the general trend of prestorage treated fruits showed lower TSS and higher TA throughout the storage period compared to the control at both 0 and 5°C (Figures 3A–D). The average BAR of the Madoka peach at harvest was 17.29. In this study, the BAR attained 24.41, 18.53, and 18.27 for the control, 1-MCP, and high CO_2_ treatments, respectively, in the case of storage at 0°C on the 28th day of storage. Similarly, the higher BAR values of 28.69, 28.89, and 21.77 were recorded at 5°C from the control, 1-MCP, and high CO_2_ treatments, respectively, on the 28th day of storage (Figures 3E,F). In general, the storage at both 0 and 5°C either maintained or increased the BAR, regardless of treatments.

**FIGURE 4:**
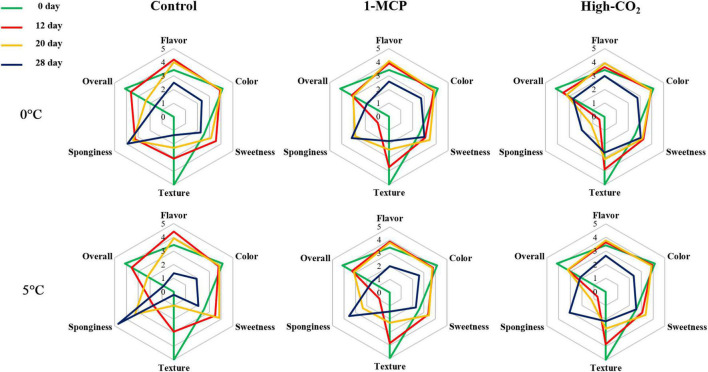
Sensory qualities of Madoka peach fruit at harvest (0 day) and during storage up to 28 day at 0 and 5°C with prestorage high CO_2_ and 1-MCP treatments or without treatment (control). Data are presented as the mean value of the subjective scale from bad (1) to excellent (5) made by 10 graduate students as panelists.

As shown in Figure 4, the overall sensory evaluation results were above 3 up to the 20th day storage for both high CO_2_ and 1-MCP treatments at both 0 and 5°C. However, the overall sensory evaluation results were above 3 up to the 12th day for the control fruits at both 0 and 5°C.

### Changes in the Total Phenolics and Flavonoids

In this study, significant differences between the treatments were detected and we observed a total phenolics ranging 2.81–3.67 and 3.18–3.47 mg g^–1^ dry weight at 0 and 5°C, respectively (Table 1). We also found total flavonoids ranging from 2.28 to 2.59 and 2.23 to 2.70 mg g^–1^ dry weight at 0 and 5°C, respectively. Regarding the treatments, both prestorage high CO_2_ and 1-MCP treatments were effective as compared to the control in maintaining total phenolics and flavonoids contents up to 20 days of storage at both 0 and 5°C (Table 1). Total phenolics content of 3.50 mg g^–1^ was recorded at harvest and maintained in both the control and treatments on the 12th day at 0°C. However, the trend of control showed a decrease in total phenolics in the case of storage at 5°C. On the other hand, high CO_2_ maintained total flavonoids at 0°C up to the 20th day but there was no clear trend for the total flavonoids at 5°C. Taken together, storage of Madoka peach at 0°C maintained total phenolics and flavonoids content, regardless of prestorage treatment with high CO_2_ and 1-MCP (Table 1).

**TABLE 1:** Changes in total phenolics, flavonoids, DPPH radical scavenging capacity, Trolox equivalent antioxidant capacity (ABTS), FRAP, and reducing power of Madoka peach fruit at harvest and during storage at 0 and 5°C with or without prestorage treatment of CO_2_ and 1-MCP.

Storage temperatures	Parameters	Storage days						
		At harvest	12			20		
			Control	CO_2_	1-MCP	Control	CO_2_	1-MCP
0°C	Total phenolics (mg GAE g^–1^)	3.50 ± 0.06a	3.58 ± 0.36a	3.56 ± 0.47a	3.67 ± 0.58a	3.24 ± 0.50ab	3.24 ± 0.02ab	2.81 ± 0.02b
	Total flavonoids (mg QE g^–1^)	2.38 ± 0.08ab	2.30 ± 0.07b	2.40 ± 0.18ab	2.47 ± 0.07ab	2.53 ± 0.25ab	2.59 ± 0.08a	2.28 ± 0.13b
	DPPH (%)	28.48 ± 0.64a	27.68 ± 0.61a	26.29 ± 1.03b	26.52 ± 0.93ab	23.79 ± 0.76cd	24.40 ± 0.49c	22.67 ± 0.72d
	ABTS (%)	13.77 ± 0.24a	14.51 ± 1.19a	12.85 ± 0.75ab	13.25 ± 1.11ab	12.70 ± 0.90ab	13.28 ± 1.69ab	12.52 ± 0.41b
	FRAP (Absorbance)	0.089 ± 0.001ab	0.090 ± 0.007a	0.085 ± 0.001a	0.086 ± 0.001a	0.081 ± 0.001b	0.085 ± 0.001b	0.079 ± 0.001b
	Reducing power (Absorbance)	0.168 ± 0.002ab	0.166 ± 0.001a	0.167 ± 0.001a	0.168 ± 0.001a	0.167 ± 0.003a	0.167 ± 0.002a	0.166 ± 0.001a
5°C	Total phenolics (mg GAE g^–1^)	3.50 ± 0.06a	3.40 ± 0.21b	3.18 ± 0.03e	3.47 ± 0.05a	3.27 ± 0.03d	3.34 ± 0.02c	3.38 ± 0.01bc
	Total flavonoids (mg QE g^–1^)	2.38 ± 0.08bc	2.54 ± 0.19ab	2.23 ± 0.18c	2.38 ± 0.10bc	2.52 ± 0.18ab	2.70 ± 0.05a	2.50 ± 0.04ab
	DPPH (%)	28.48 ± 0.64a	25.78 ± 0.08b	24.62 ± 0.21b	25.17 ± 0.45b	23.15 ± 0.69c	23.09 ± 0.81c	23.46 ± 1.18bc
	ABTS (%)	13.77 ± 0.24c	15.01 ± 0.64a	14.36 ± 0.24ab	14.40 ± 0.24a	13.13 ± 0.81c	13.16 ± 0.64bc	13.90 ± 0.25bc
	FRAP (Absorbance)	0.089 ± 0.001a	0.088 ± 0.002a	0.086 ± 0.001b	0.087 ± 0.001ab	0.086 ± 0.002b	0.087 ± 0.001ab	0.087 ± 0.001ab
	Reducing power (Absorbance)	0.168 ± 0.002a	0.166 ± 0.001a	0.162 ± 0.001b	0.167 ± 0.001a	0.165 ± 0.001a	0.163 ± 0.001b	0.167 ± 0.002a

*Results are presented as the mean ± SD from triplicate independent values. Means with different letters within the same row are significantly different at p < 0.05. All data were detected from 1 mg ml^–1^ peach samples on dry weight basis.*

### Changes in Antioxidant Activities

In this study, we analyzed the antioxidant activities of prestorage high CO_2_ and 1-MCP-treated Madoka peach fruit at harvest, on the 12th and 20th days during storage at 0 and 5°C by using DPPH, ABTS, FRAP, and reducing power assays as shown in Table 1. The results showed significant differences between the treatments, and the 12th day displayed higher antioxidant activities than the 20th day in DPPH and ABTS assays at both 0 and 5°C. The FRAP and reducing power assays also showed either maintaining or reducing trends, irrespective of treatments, at both 0 and 5°C on the 20th day compared to the absorbance values at harvest and on the 12th day. Moreover, the antioxidant activities of prestorage treated Madoka peach fruit were statistically higher or similar to the control fruits up to the 20th day at both 0 and 5°C.

## Discussion

Peach (*P. persica* L.) is one of the highly perishable fruits that deteriorates quickly at room temperature. Thus, cold storage is a common strategy for slowing the ripening process and extending the shelf life of peach fruit (3). Contrarily, low-temperature storage may result in improper ripening and CI in susceptible cultivars, such as Madoka (3). The prestorage treatments, such as 1-MCP and high CO_2_, have been shown to reduce CI (6, 18). Hence, we treated Madoka peach fruit with high CO_2_ and 1-MCP before storing them at 0 and 5°C. The treated fruits were compared to the control fruits to investigate CI, the changes in postharvest quality and storability, and the antioxidant properties.

The difference in the effectiveness of high CO_2_ and 1-MCP in reducing CI and delaying ripening can be explained by the difference in their diffusion. Graham’s law states that heavier gases diffuse more slowly than lighter gases (19). The 1-MCP is heavier than CO_2_ implying that 1-MCP diffuses more slowly than CO_2_ which would affect its action on the quality and storability of peach fruit. In addition, the surface of Madoka peach fruit is covered with dense hairs, and previous studies on peach fruit pubescence have revealed that the surface of the peach fruit is covered with trichomes and cuticles that may serve as protective layers against biotic and abiotic stresses (20, 21). So, the application of 1-MCP without the removal of the external hairs may also reduce its effectiveness in cultivars, such as Madoka, as it could be a barrier to gas diffusion (20). Pervitasari et al. (20) reported that hydrocooling before 1-MCP application improved the effectiveness of 1-MCP by removing most of the hair on “Mibaekdo” peach fruit. Regarding the differences among the storage temperatures, similar to our results, the previous studies have reported that prolonged storage at low-temperature results in CI in susceptible peach cultivars during or after cold storage (3, 5), particularly with storage temperatures in the range 2–8°C as compared to those stored at 0°C (7). According to Lee (6), moderately and severely injured fruits with a CI index greater than 20% are not commercially acceptable due to injury to the mesocarp surface on opposite sides of the stone. Hence, based on our results for CI index, Madoka peaches are acceptable for marketing up to 20 and 16 days of storage at 0 and 5°C, respectively, while treated fruits could be marketable up to 4 weeks of storage.

Softening is an important indicator of ripening that influences peach fruit storage and distribution (3). Cognizant of this, firmness data for Madoka peach fruit were collected during the experiment to compare the prestorage high CO_2_ and 1-MCP-treated fruits with the control. Firmer fruits in high CO_2_ treatment at 0°C can be explained by reduction of metabolic processes due to low temperature and treating peach fruit with high CO_2_ before the storage could reduce the respiration rate and inhibit the effect of ethylene (22, 23). Similarly, firmer fruit in 1-MCP-treated fruits at 0°C can be explained by lowering of metabolic processes due to low temperature and blocking of ethylene receptors by 1-MCP (22, 24). Choi et al. (3) reported that the cell wall of peach fruit is degraded or modified during the ripening process. Pectin may be solubilized and depolymerized during fruit ripening, which contributes to middle lamella erosion and primary cell wall disintegration, resulting in softening and a decrease in firmness (25). In this study, the reduced rate of solubilization of pectin in prestorage high CO_2_ and 1-MCP-treated fruits could be attributed to the reduced activity of cell wall hydrolytic enzymes as evidenced by PG activity (Figures 1G,H). In agreement with our result, Choi et al. (3) also reported reduced PG activity in prestorage high CO_2_ and 1-MCP-treated fruits as compared to the control. The gradual trends of PG activity in the control and treated groups at 0°C can be explained by lowering of enzymatic activity due to low temperature.

The lower respiration rate in high CO_2_ treatment at 0°C could be due to the downregulation of a gene that encodes 1-aminocyclopropane-1-carboxylate oxidase (ACCO), an enzyme that catalyzes the biosynthesis of ethylene (3). Earlier peaks in ethylene production at 5°C could be the reason to trigger the climacteric rise of respiration and attain the peaks on the 20th day of storage.

**FIGURE 5:**
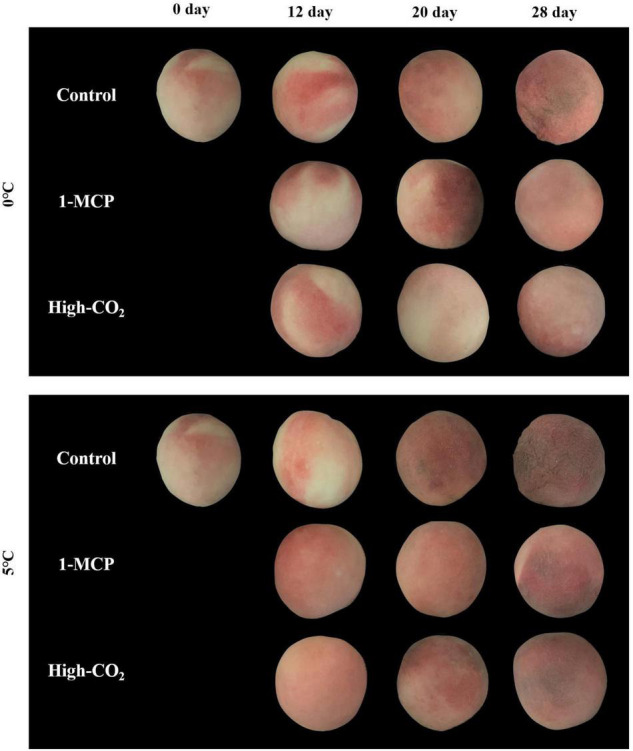
Madoka peach fruit at harvest (0 day) and during storage up to 28 days at 0 and 5°C with or without prestorage treatment of 1-MCP and high CO_2_.

Leonel et al. (26) reported the BAR ranging from 14 to 35 after evaluating five peach cultivars for two growing cycles, implying cultivar and growing conditions were dependent on BAR values of peach fruit. Tilahun et al. (22) and Helyes et al. (27)reported that higher BAR means better and harmonious flavor during their evaluation of tomatoes. Therefore, the preference for Madoka peach fruit starting from day 0 (at harvest) as evidenced by the results of the sensory qualities, such as flavor, color, sweetness, and overall sensory values (Figure 4), could be attributed to its high BAR. In addition to the BAR, the appearance (Figure 5) and the texture as evidenced by firmness and firmness-related parameters (Figure 1) could play a paramount role in overall preference by consumers.

Secondary metabolites are compounds that aid in the defense, aroma, and pigmentation of fruits (10). Secondary metabolites, such as phenolic and terpenoid compounds, are responsible for the color, organoleptic, and nutritional properties of fruits (28). In agreement with this study, Andreotti et al. (29) reported an average of 3 mg g^–1^ dry weight (DW) total phenolics in pulp tissues of yellow “Stark Red Gold” nectarine at ripening during their evaluation of phenolic compounds in peach cultivars at harvest and during fruit development. Similarly, Mokrani and Madani (30) reported 3.63 mg g^–1^ DW total phenolics as the highest value during their study on the effect of solvent, time, and temperature on the extraction of phenolic compounds from peach fruit. However, our total flavonoids data in the current study (2.23–2.70 mg g^–1^ DW) are higher than the value (0.57 mg g^–1^ DW) reported by Mokrani and Madani (30) as the highest total flavonoids in their study. The difference could be attributed to the variation in the cultivar and growing conditions. From the above results, the observed total phenolics and flavonoids content was also maintained due to prestorage high CO_2_ and 1-MCP treatments, which could be the main reason for maintaining the antioxidant activity. Antioxidant activity assays showed statistically higher or similar antioxidant activities of both high CO_2_ and 1-MCP-treated Madoka peach fruit up to the 20th day at both 0 and 5°C compared to the control, implying the effectiveness of both high CO_2_ and 1-MCP in maintaining the antioxidant activity of peach fruit during cold storage. In agreement with this study, Tilahun et al. (22) reported the effectiveness of prestorage high CO_2_ and 1-MCP treatments combined with modified atmosphere packaging in maintaining the antioxidant activity of “Unicorn” cherry tomato.

## Conclusion

The results of CI index, firmness, and firmness-related parameters (pectin content and PG activity) revealed that both high CO_2_ and 1-MCP treatments were effective in reducing CI and delaying the ripening process of Madoka peach, and storage at 0°C showed better results than 5°C. However, based on the sensory evaluation results, the treated and control fruits were acceptable for marketing up to 20 and 12 days of storage, respectively, in both storage conditions. After deciding on fruit marketability based on the combined objective postharvest quality parameters and subjective sensory qualities, we analyzed the changes in total phenolics, flavonoids, and antioxidant activities at harvest, on the 12th and 20th day of cold storage. Storage of Madoka peach at 0°C maintained total phenolics, flavonoids, and antioxidant activities, regardless of prestorage treatment with high CO_2_ and 1-MCP. In summary, storing Madoka peach fruit at 0°C after treating it with 30% CO_2_ for 6 h or 0.5 μL L^–1^ 1-MCP for 24 h reduces CI, prolongs storability, and maintains sensory quality and antioxidant properties.

## Data Availability Statement

The raw data supporting the conclusions of this article will be made available by the authors, without undue reservation.

## Author Contributions

ST and CJ contributed to conceptualization, methodology, and supervision. HC, MB, and MJ contributed to data curation. ST and HC contributed to software and formal analysis. CJ contributed to resources and funding acquisition. ST and MJ contributed to original draft preparation. JH contributed to review and editing. HC contributed to project administration. All authors have read and agreed to the published version of the manuscript.

## Conflict of Interest

The authors declare that the research was conducted in the absence of any commercial or financial relationships that could be construed as a potential conflict of interest.

## Publisher’s Note

All claims expressed in this article are solely those of the authors and do not necessarily represent those of their affiliated organizations, or those of the publisher, the editors and the reviewers. Any product that may be evaluated in this article, or claim that may be made by its manufacturer, is not guaranteed or endorsed by the publisher.
